# Supplementary material for: Unveiling the Bioactive Potential of the Invasive Jellyfish Phyllorhiza punctata Through Integrative Transcriptomic and Proteomic Analyses
Source: Biomolecules. 2025 Aug 4;15(8):1121. doi: 10.3390/biom15081121 (PMC12383608; doi:10.3390/biom15081121)
Supplement: Supplementary file 1 [file biomolecules-15-01121-s001.zip › Suplementary Material/FileS3/PEPFOLD4/XP_065065638_8308/clusters.html]

 PEP-FOLD clusters


Download archive of all models  here   

|  |  |  |
| --- | --- | --- |
| # | file | sOPEP |
| # cluster 1 - Size: 1 |  |  |
|  | XP\_065065638\_8308-model1 | -65.664 |
| # cluster 2 - Size: 1 |  |  |
|  | XP\_065065638\_8308-model2 | -65.0598 |
| # cluster 3 - Size: 1 |  |  |
|  | XP\_065065638\_8308-model3 | -64.8838 |
| # cluster 4 - Size: 1 |  |  |
|  | XP\_065065638\_8308-model4 | -63.5776 |
| # cluster 5 - Size: 1 |  |  |
|  | XP\_065065638\_8308-model5 | -63.5713 |
| # cluster 6 - Size: 1 |  |  |
|  | XP\_065065638\_8308-model6 | -61.5104 |
| # cluster 7 - Size: 1 |  |  |
|  | XP\_065065638\_8308-model7 | -60.0203 |
| # cluster 8 - Size: 1 |  |  |
|  | XP\_065065638\_8308-model8 | -59.9484 |
| # cluster 9 - Size: 2 |  |  |
|  | XP\_065065638\_8308-model9 | -58.5622 |
|  | XP\_065065638\_8308-model9.1 | -57.733 |
| # cluster 10 - Size: 2 |  |  |
|  | XP\_065065638\_8308-model10 | -58.4245 |
|  | XP\_065065638\_8308-model10.1 | -49.0834 |
| # cluster 11 - Size: 1 |  |  |
|  | XP\_065065638\_8308-model11 | -58.2466 |
| # cluster 12 - Size: 1 |  |  |
|  | XP\_065065638\_8308-model12 | -58.0575 |
| # cluster 13 - Size: 1 |  |  |
|  | XP\_065065638\_8308-model13 | -57.9991 |
| # cluster 14 - Size: 1 |  |  |
|  | XP\_065065638\_8308-model14 | -57.589 |
| # cluster 15 - Size: 1 |  |  |
|  | XP\_065065638\_8308-model15 | -57.4157 |
| # cluster 16 - Size: 1 |  |  |
|  | XP\_065065638\_8308-model16 | -57.2507 |
| # cluster 17 - Size: 1 |  |  |
|  | XP\_065065638\_8308-model17 | -56.8589 |
| # cluster 18 - Size: 1 |  |  |
|  | XP\_065065638\_8308-model18 | -55.697 |
| # cluster 19 - Size: 1 |  |  |
|  | XP\_065065638\_8308-model19 | -55.4257 |
| # cluster 20 - Size: 1 |  |  |
|  | XP\_065065638\_8308-model20 | -55.2977 |
| # cluster 21 - Size: 1 |  |  |
|  | XP\_065065638\_8308-model21 | -54.7922 |
| # cluster 22 - Size: 1 |  |  |
|  | XP\_065065638\_8308-model22 | -54.5297 |
| # cluster 23 - Size: 1 |  |  |
|  | XP\_065065638\_8308-model23 | -53.9641 |
| # cluster 24 - Size: 1 |  |  |
|  | XP\_065065638\_8308-model24 | -53.8965 |
| # cluster 25 - Size: 1 |  |  |
|  | XP\_065065638\_8308-model25 | -53.8295 |
| # cluster 26 - Size: 1 |  |  |
|  | XP\_065065638\_8308-model26 | -53.7118 |
| # cluster 27 - Size: 1 |  |  |
|  | XP\_065065638\_8308-model27 | -53.4488 |
| # cluster 28 - Size: 2 |  |  |
|  | XP\_065065638\_8308-model28 | -53.4483 |
|  | XP\_065065638\_8308-model28.1 | -48.4545 |
| # cluster 29 - Size: 1 |  |  |
|  | XP\_065065638\_8308-model29 | -53.1906 |
| # cluster 30 - Size: 1 |  |  |
|  | XP\_065065638\_8308-model30 | -53.1596 |
| # cluster 31 - Size: 1 |  |  |
|  | XP\_065065638\_8308-model31 | -53.079 |
| # cluster 32 - Size: 1 |  |  |
|  | XP\_065065638\_8308-model32 | -53.0177 |
| # cluster 33 - Size: 1 |  |  |
|  | XP\_065065638\_8308-model33 | -52.7112 |
| # cluster 34 - Size: 1 |  |  |
|  | XP\_065065638\_8308-model34 | -52.6641 |
| # cluster 35 - Size: 1 |  |  |
|  | XP\_065065638\_8308-model35 | -52.6195 |
| # cluster 36 - Size: 1 |  |  |
|  | XP\_065065638\_8308-model36 | -52.5429 |
| # cluster 37 - Size: 1 |  |  |
|  | XP\_065065638\_8308-model37 | -52.4848 |
| # cluster 38 - Size: 1 |  |  |
|  | XP\_065065638\_8308-model38 | -52.0344 |
| # cluster 39 - Size: 1 |  |  |
|  | XP\_065065638\_8308-model39 | -51.882 |
| # cluster 40 - Size: 1 |  |  |
|  | XP\_065065638\_8308-model40 | -51.4574 |
| # cluster 41 - Size: 1 |  |  |
|  | XP\_065065638\_8308-model41 | -51.4502 |
| # cluster 42 - Size: 1 |  |  |
|  | XP\_065065638\_8308-model42 | -50.8805 |
| # cluster 43 - Size: 1 |  |  |
|  | XP\_065065638\_8308-model43 | -50.8229 |
| # cluster 44 - Size: 1 |  |  |
|  | XP\_065065638\_8308-model44 | -50.7159 |
| # cluster 45 - Size: 1 |  |  |
|  | XP\_065065638\_8308-model45 | -50.7024 |
| # cluster 46 - Size: 1 |  |  |
|  | XP\_065065638\_8308-model46 | -50.5768 |
| # cluster 47 - Size: 1 |  |  |
|  | XP\_065065638\_8308-model47 | -50.3993 |
| # cluster 48 - Size: 1 |  |  |
|  | XP\_065065638\_8308-model48 | -49.9813 |
| # cluster 49 - Size: 1 |  |  |
|  | XP\_065065638\_8308-model49 | -49.9081 |
| # cluster 50 - Size: 1 |  |  |
|  | XP\_065065638\_8308-model50 | -49.8473 |
| # cluster 51 - Size: 1 |  |  |
|  | XP\_065065638\_8308-model51 | -49.7834 |
| # cluster 52 - Size: 1 |  |  |
|  | XP\_065065638\_8308-model52 | -49.7357 |
| # cluster 53 - Size: 1 |  |  |
|  | XP\_065065638\_8308-model53 | -49.6606 |
| # cluster 54 - Size: 1 |  |  |
|  | XP\_065065638\_8308-model54 | -49.4814 |
| # cluster 55 - Size: 1 |  |  |
|  | XP\_065065638\_8308-model55 | -49.456 |
| # cluster 56 - Size: 1 |  |  |
|  | XP\_065065638\_8308-model56 | -49.3253 |
| # cluster 57 - Size: 1 |  |  |
|  | XP\_065065638\_8308-model57 | -49.2746 |
| # cluster 58 - Size: 1 |  |  |
|  | XP\_065065638\_8308-model58 | -49.1598 |
| # cluster 59 - Size: 2 |  |  |
|  | XP\_065065638\_8308-model59 | -49.1149 |
|  | XP\_065065638\_8308-model59.1 | -47.9406 |
| # cluster 60 - Size: 1 |  |  |
|  | XP\_065065638\_8308-model60 | -49.0875 |
| # cluster 61 - Size: 1 |  |  |
|  | XP\_065065638\_8308-model61 | -49.0872 |
| # cluster 62 - Size: 1 |  |  |
|  | XP\_065065638\_8308-model62 | -48.9895 |
| # cluster 63 - Size: 1 |  |  |
|  | XP\_065065638\_8308-model63 | -48.7728 |
| # cluster 64 - Size: 1 |  |  |
|  | XP\_065065638\_8308-model64 | -48.6182 |
| # cluster 65 - Size: 1 |  |  |
|  | XP\_065065638\_8308-model65 | -48.2684 |
| # cluster 66 - Size: 1 |  |  |
|  | XP\_065065638\_8308-model66 | -48.2531 |
| # cluster 67 - Size: 1 |  |  |
|  | XP\_065065638\_8308-model67 | -48.0648 |
| # cluster 68 - Size: 1 |  |  |
|  | XP\_065065638\_8308-model68 | -47.8847 |
| # cluster 69 - Size: 1 |  |  |
|  | XP\_065065638\_8308-model69 | -47.6056 |
| # cluster 70 - Size: 1 |  |  |
|  | XP\_065065638\_8308-model70 | -47.5651 |
| # cluster 71 - Size: 1 |  |  |
|  | XP\_065065638\_8308-model71 | -47.528 |
| # cluster 72 - Size: 1 |  |  |
|  | XP\_065065638\_8308-model72 | -47.4875 |
| # cluster 73 - Size: 1 |  |  |
|  | XP\_065065638\_8308-model73 | -47.4546 |
| # cluster 74 - Size: 1 |  |  |
|  | XP\_065065638\_8308-model74 | -47.0652 |
| # cluster 75 - Size: 1 |  |  |
|  | XP\_065065638\_8308-model75 | -46.8423 |
| # cluster 76 - Size: 1 |  |  |
|  | XP\_065065638\_8308-model76 | -46.5614 |
| # cluster 77 - Size: 1 |  |  |
|  | XP\_065065638\_8308-model77 | -46.3267 |
| # cluster 78 - Size: 1 |  |  |
|  | XP\_065065638\_8308-model78 | -46.203 |
| # cluster 79 - Size: 1 |  |  |
|  | XP\_065065638\_8308-model79 | -46.1845 |
| # cluster 80 - Size: 2 |  |  |
|  | XP\_065065638\_8308-model80 | -45.6598 |
|  | XP\_065065638\_8308-model80.1 | -43.7846 |
| # cluster 81 - Size: 1 |  |  |
|  | XP\_065065638\_8308-model81 | -45.5029 |
| # cluster 82 - Size: 1 |  |  |
|  | XP\_065065638\_8308-model82 | -45.3176 |
| # cluster 83 - Size: 1 |  |  |
|  | XP\_065065638\_8308-model83 | -45.0439 |
| # cluster 84 - Size: 1 |  |  |
|  | XP\_065065638\_8308-model84 | -44.982 |
| # cluster 85 - Size: 1 |  |  |
|  | XP\_065065638\_8308-model85 | -44.7476 |
| # cluster 86 - Size: 1 |  |  |
|  | XP\_065065638\_8308-model86 | -43.6262 |
| # cluster 87 - Size: 1 |  |  |
|  | XP\_065065638\_8308-model87 | -43.4004 |
| # cluster 88 - Size: 1 |  |  |
|  | XP\_065065638\_8308-model88 | -42.6767 |
| # cluster 89 - Size: 1 |  |  |
|  | XP\_065065638\_8308-model89 | -41.9699 |
| # cluster 90 - Size: 1 |  |  |
|  | XP\_065065638\_8308-model90 | -41.3768 |
| # cluster 91 - Size: 1 |  |  |
|  | XP\_065065638\_8308-model91 | -41.2712 |
| # cluster 92 - Size: 1 |  |  |
|  | XP\_065065638\_8308-model92 | -40.8081 |
| # cluster 93 - Size: 1 |  |  |
|  | XP\_065065638\_8308-model93 | -40.1669 |
| # cluster 94 - Size: 1 |  |  |
|  | XP\_065065638\_8308-model94 | -39.2108 |
| # cluster 95 - Size: 1 |  |  |
|  | XP\_065065638\_8308-model95 | -37.9633 |
|  |  |  |
